# Robotic prostatectomy: an update on functional and oncologic outcomes

**DOI:** 10.3332/ecancer.2013.355

**Published:** 2013-09-26

**Authors:** Gabriele Cozzi, Elisa De Lorenzis, Carlotta Palumbo, Pietro Acquati, Giancarlo Albo, Paolo dell’Orto, Angelica Grasso, Bernardo Rocco

**Affiliations:** Department of Specialist Surgical Sciences, University of Milan, Urology Department, Fondazione IRCCS Ca’ Granda Ospedale Maggiore Policlinico, Milan, Italy

**Keywords:** robotic surgery, prostate cancer, prostatectomy, incontinence, impotence

## Abstract

Since the first procedure performed in 2000, robotic-assisted radical prostatectomy (RARP) has been rapidly gaining increasing acceptance from both urologists and patients. Today, RARP is the dominant treatment option for localised prostate cancer (PCa) in the United States, despite the absence of any prospective randomised trial comparing RARP with other procedures.

Robotic systems have been introduced in an attempt to reduce the difficulty involved in performing complex laparoscopic procedures and the related steep learning curve. The recognised advantages of this kind of minimally invasive surgery are three-dimensional (3D) vision, ten-fold magnification, Endowrist technology with seven degrees of freedom, and tremor filtration.

In this article, we examine this technique and report its functional (in terms of urinary continence and potency) and oncologic results. We also evaluate the potential advantages of RARP in comparison with open and laparoscopic procedures.

## Introduction

Since the initial description of laparoscopic radical prostatectomy (LRP) by Schuessler *et al* in 1992 [1], the laparoscopic procedure has been standardised by Guillonneau and Vallancien [2] and others [3]; even if LRP replaced open retropubic prostatectomy (ORP) in some centres, it is universally considered a challenging procedure. In fact, besides perfect knowledge of the local anatomy, LRP requires complex laparoscopic skills, such as endoscopic suturing and intracorporeal knotting, which make the learning curve particularly steep.

In 1999, the da Vinci Surgical System^®^ was developed by Intuitive Surgical Inc. (Mountain View, California, United States), while, in 2000, Binder *et al* described the first ten robotic-assisted radical prostatectomy (RARP) procedures [4]. Many features of this robotic surgical system were very useful in performing this kind of surgery: (i) the improvement in visualisation by the InSite Vision System, through three-dimensional (3D) vision, ten-fold magnification, and infinitely variable positioning of the 30° endoscope by the surgeon; (ii) the facilitation of handling of laparoscopic tools by the 3D vision, which allows to dissect, suture, and tie knots endoscopically much as would be possible in open surgery; and (iii) the better control of surgical instruments and camera positioning at the console.

The main disadvantages observed at that time were the limited choice of laparoscopic tools and the considerable cost.

## The adoption of RARP

At present, radical prostatectomy (RP) represents the standard for long-term cure of localised prostate cancer (PCa), with cancer-specific survival approaching 95% at 15 years after RP [5]. Since the first procedure performed by Binder in May 2000 [4], RARP, performed using the da Vinci surgical system, has been rapidly accepted as a safe and efficacious treatment option for localised PCa [6]. RARP is currently the leading urologic use of the da Vinci system, and more than 80% of the RPs performed in the United States in 2011 were done by robotic-assisted surgery [7].

RARP can be performed either through a transperitoneal or through a subperitoneal approach, with more precision and choices for dissection, thanks to the 3D vision of the system [7].

### Indications for RARP

Indications for RARP are the same as those for ORP and LRP. According to the American Urological Association (AUA) Guidelines 2007 (reviewed and validity confirmed 2011), low-, intermediate-, and high-risk patients with localised PCa could undergo RARP [8]. The European Association of Urology (EAU) Guidelines 2011 identify four categories of patients who should undergo RALP: patients with low- and intermediate-risk localised PCa and a life expectancy >10 years; patients with stage T1a disease and a life expectancy of >15 years or Gleason score (GS) 7; selected patients with low-volume high-risk localised PCa; highly selected patients with very high-risk localised PCa (cT3b-T4 N0 or any T N1) in the context of multimodal treatment [9].

Further, the Pasadena Consensus Panel (PCP) identified some patient subgroups who should be treated by an ‘experienced’ surgeon, such as obese patients [body mass index (BMI) > 30], patients with prostate volume > 70 cm^3^, patients with previous transurethral resection of prostate (TURP) or other surgery for benign prostatic hyperplasia (BPH), patients with large median lobe, high-risk patients requiring extended pelvic lymph node dissection, and patients with previous pelvic surgery [10]. Only very experienced surgeons should perform salvage RARP after radiation therapy, cryotherapy, or high-intensity focused ultrasound (HIFU) [11].

Recent studies showed that RARP can be performed safely without discontinuing low-dose aspirin, without significant increases in blood loss, transfusion rates, and postoperative haemorrhagic complications [12].

### Robotic-assisted lymph node dissection

The lymph node drainage of the prostate appears to be in the following order: external and obturator (38%), internal iliac (25%), common iliac (16%), para-aortic/caval (12%), presacral (8%), and inguinal (1%) [13]. As before, indications for lymph dissection during RARP are the same as those for ORP: patients with intermediate-risk PCa (cT2a and/or PSA 10–20 ng/mL and/or biopsy GS of 7), high-risk PCa (>cT2b and/or PSA > 20 ng/mL and/or GS ≥ 8), or patients with ≥7% likelihood of having node metastases according to the available nomograms [10].

Freicke *et al* [14], Ham *et al* [15], and Menon *et al* [16] reported the feasibility of an extended lymph node dissection in course of RARP, including external iliac, internal iliac, and obturator lymph nodes. They obtained mean numbers of nodes ranging from 12 to 19 and positive node rates ranging from 11% to 24%, according to the different patient characteristics [17].

Chung *et al* [18] compared transperitoneal and extraperitoneal limited dissection, showing a similar lymph node yield with a slightly higher risk of postoperative lymphocoeles for the extraperitoneal approach.

### Oncologic results after RARP

Although 13 years have passed since the first procedure, only a few centres have been performing RARP for more than 5 years. Thus, long-term data regarding biochemical recurrence (BCR) of PCa after RARP are still sparse. Data from the most detailed available RARP series show BCR-free survival estimates of 95.1%, 90.6%, 86.6%, and 81.0% at 1, 3, 5, and 7 years after RARP, respectively (median follow-up: 5 years) [10].

A surrogate oncologic outcome can be identified in the positive surgical margin (PSM) rates. PSMs are defined as presence of tumour at the inked margin of the prostatectomy specimen, and they are considered a risk factor for disease progression [10]. A large population-based study showed that patients with PSMs had a 1.7-fold higher risk of death compared with those without [19]. A recent systematic review of oncologic outcomes after RARP showed that the average rate of PSMs in pT2 disease is 8%–10%, while in pT3 disease is about 37% [17]. PSMs in pT2 disease can be considered for the most part iatrogenic and hence potentially avoidable [20].

In 2013, Silberstein *et al* published a study comparing early oncologic outcomes of 961 ORP and 493 RARP performed by experienced surgeons in a high volume centre. Despite a short follow-up (1 year), they found that RARP was not associated with lower rates of BCR-free survival or higher rates of PSMs. The same findings were confirmed for higher-risk patients receiving RARP when emphasis is placed on strict adherence to oncological surgical principles [21].

### Continence outcomes after RARP

While incontinence and impotence are the two chief drawbacks of RP [22], incontinence seems to be the problem that troubles patients most, even if its incidence is inferior to that of impotence. According to the EAU Guidelines 2011, incontinence persists 1 year after RP in 7.7% of cases [9], while the AUA Guidelines 2007 (reviewed and validity confirmed 2011) report post-RP incontinence rates ranging from 3% to 74% [8].

The International Continence Society defined incontinence as ‘the complaint of any involuntary leakage of urine’ [23, 24]. Stress incontinence is the most frequently observed type of incontinence after RP, even if a considerable number of patients present a mixed urge and stress syndrome.

Sphincter dysfunction is mainly a result of injury to the sphincter mechanism during prostatic surgery; considering this mechanism, incontinence is usually associated with abdominal pressure increase. In the most severe cases, it can be gravitational [25].

The PCP identified some risk factors for urinary incontinence following RARP, such as increased age, obesity, short membranous urethral length on both preoperative and postoperative endorectal magnetic resonance imaging (MRI), post-RARP anastomotic strictures, low institutional and/or surgeon caseload, resection of the neurovascular bundles (NVBs) and/or of the bladder neck, large prostate volume [10].

The prevalence of urinary incontinence after RARP ranges from 4% to 31%. These outcomes can be influenced by preoperative patient characteristics, surgeon experience, surgical technique, and methods used to collect and report data [26].

Data from a recent meta-analysis showed a statistically significant advantage in favour of RARP in comparison with both RRP and LRP in terms of 12-month urinary continence recovery [26].

Many surgical techniques have been proposed in order to reduce the occurrence of incontinence and to shorten the time to continence. Between them, in 2001, Rocco *et al* described a surgical technique whose aim was the reconstruction of the posterior musculofascial plate after RP in order to improve early return to urinary continence [27]. In 2007, the application of this technique to transperitoneal LRP was described [28]. In 2012, Rocco *et al* conducted a systematic review of comparative studies analysing the influence of reconstruction of the posterior aspect of the rhabdosphincter during ORP, LRP, and RARP. The cumulative analysis of comparative studies showed that reconstruction of the posterior musculofascial plate improves early return of continence within the first 30 days after RP, while continence rates 90 days after surgery are not affected by the use of the reconstruction technique [29].

### Potency after RARP

The NVBs were first described in 1982 by Walsh and Donker [30]. These authors demonstrated that erectile dysfunction following RP occurred secondary to injury to the cavernosal nerves (CNs), a group of parasympathetic nerves originating from the pelvic plexus and running together with arteries and veins (capsular vessels of the prostate) on a prominent NVB on the posterolateral aspect of the prostate and eventually ending in the corpus cavernosum of the penis.

Further studies about the distribution of nerves within the NVB demonstrated that these nerves are organised into three functional compartments, in which the CNs are located on the anteromedial aspect of the NVB closest to the prostate. Other nerves within the NVB located laterally and inferiorly to the CN innervate the levator ani muscle and rectum, respectively [31, 32].

According to the PCP, a maximum preservation of CNs (full nerve sparing) can be obtained by following the plane between the prostatic capsule and the multilayer tissue of the prostatic fascia. This kind of nerve sparing is recommended for sexually active and functional men without comorbidities and limited-risk disease. Partial nerve sparing, obtained following the planes within the multilayer tissue of the prostatic fascia, is recommended for preoperative potent men without comorbidities and intermediate- or high-risk localised disease, while patients with erectile dysfunction and/or comorbidities, or not interested in sexual activity, should undergo minimal nerve sparing, which is the preservation of CNs running at the posterolateral surface of the prostate. When the disease is clearly extraprostatic, patients should undergo a non-nerve-sparing surgery [10].

A recent systematic review of the literature by Ficarra *et al* showed that, for patients undergoing RARP, relevant predictors of postoperative potency are age at surgery, baseline erectile function, and presence of comorbidities. They reported that nerve-sparing RARP was associated with an incidence of 12- and 24-month erectile dysfunction ranging from 10% to 46% and from 6% to 37%, respectively.

## Conclusion

In conclusion, the authors demonstrated, for the first time, a significant advantage in favour of RARP in comparison with ORP in terms of 12-month potency rates [33].
